# Increased Gene Expression of RUNX2 and SOX9 in Mesenchymal Circulating Progenitors Is Associated with Autophagy during Physical Activity

**DOI:** 10.1155/2019/8426259

**Published:** 2019-10-15

**Authors:** L. Dalle Carbonare, M. Mottes, S. Cheri, M. Deiana, F. Zamboni, D. Gabbiani, F. Schena, G. L. Salvagno, G. Lippi, M. T. Valenti

**Affiliations:** ^1^Department of Medicine, University of Verona, Italy; ^2^Department of Neurosciences, Biomedicine and Movement Sciences, University of Verona, Italy

## Abstract

Lack of physical exercise is considered an important risk factor for chronic diseases. On the contrary, physical exercise reduces the morbidity rates of obesity, diabetes, bone disease, and hypertension. In order to gain novel molecular and cellular clues, we analyzed the effects of physical exercise on differentiation of mesenchymal circulating progenitor cells (M-CPCs) obtained from runners. We also investigated autophagy and telomerase-related gene expression to evaluate the involvement of specific cellular functions in the differentiation process. We performed cellular and molecular analyses in M-CPCs, obtained by a depletion method, of 22 subjects before (PRE RUN) and after (POST RUN) a half marathon performance. In order to prove our findings, we performed also *in vitro* analyses by testing the effects of runners' sera on a human bone marrow-derived mesenchymal stem (hBM-MSC) cell line. PCR array analyses of PRE RUN versus POST RUN M-CPC total RNAs put in evidence several genes which appeared to be modulated by physical activity. Our results showed that physical exercise promotes differentiation. Osteogenesis-related genes as RUNX2, MSX1, and SPP1 appeared to be upregulated after the run; data showed also increased levels of BMP2 and BMP6 expressions. SOX9, COL2A1, and COMP gene enhanced expression suggested the induction of chondrocytic differentiation as well. The expression of telomerase-associated genes and of two autophagy-related genes, ATG3 and ULK1, was also affected and correlated positively with MSC differentiation. These data highlight an attractive cellular scenario, outlining the role of autophagic response to physical exercise and suggesting new insights into the benefits of physical exercise in counteracting chronic degenerative conditions.

## 1. Introduction

Lack of physical exercise is considered an important risk factor for chronic diseases. On the contrary, physical exercise counteracts obesity, diabetes, bone disease, and hypertension [1] as well as other degenerative diseases [2, 3]. In addition, physical exercise plays a central role in maintaining bone quality [4]. Recently, we identified several proteins differentially expressed in runners' sera after the half marathon. Despite several negative cellular effects, such as oxidative stress, the resulting proteome modulation seems to be beneficial for the overall health [5]. We also demonstrated that running the half marathon increases the expression of the osteogenic transcription factor RUNX2 as well as the calcification process in an *in vitro* model. Furthermore, we found a reduction in the adipogenic commitment of a human bone marrow-derived mesenchymal stem cell (hBM-MSC) line treated with sera obtained from runners [5]. MSCs, together with hematopoietic, endothelial, and smooth or skeletal muscle precursors can be found in peripheral blood as circulating progenitor cells (CPCs) [6, 7]. In the past, we have described the isolation of MSC-like cells from peripheral blood (CD3^−^, CD19^−^, CD14^low^, CD45^low^, and CD34^low^), characterized by clonogenic growth and differentiation ability [8]. In addition, these mesenchymal circulating progenitor cells (M-CPCs) expressed also CD105 and CD73 markers [9]; we have investigated their involvement in various skeletal degenerative diseases [9–12]. Considering the clinical and social impact of common skeletal degenerative diseases such as osteoporosis and ostearthrosis, we were interested to evaluate the effects of physical activity on M-CPCs. Since during physical activity oxidative stress as well as pleiotropic cytokine production increases, we hypothesized that different biological processes involved in differentiation might be affected in progenitor cells. In particular, telomerase activity and autophagy, which are both involved in oxidative stress [13–15], have been suggested as biological processes affecting differentiation [16, 17]. Therefore, we hypothesized that physical activity might promote differentiation by regulating telomerase-associated genes as well autophagy.

Several studies have demonstrated that the progenitor cell number increases in peripheral blood in response to endurance and physical exercise [18–21]. Various kinds of exercise such as running, rowing, and cycling may stimulate progenitor cell mobilization; stimulation of stem cell formation sites, such as the pelvis and femur, can be considered responsible for this [19, 22]. Inflammation-related [5, 23] or adrenaline-induced factors [24] might affect progenitor cell mobilization during exercise. However, the proliferative potential of MSCs declines with age because of telomere shortening [25, 26]. In addition, telomeres can be affected by genotoxic injuries and oxidative stress [27]. Notably, short telomeres affect the proliferation, self-renewal, and commitment of progenitor cells [28] and induce alterations during their differentiation process in embryonic stem cells [16]. However, while telomere length is important to counteract mesenchymal stem cell senescence, [29], telomerase activity plays an important role in the differentiation process [30]. Increased telomerase activity due to the overexpression of human telomerase reverse transcriptase (hTERT) gene in hMSCs promotes osteoblast differentiation *in vitro* as well as heterotopic bone formation *in vivo* [31, 32]. Besides maintaining telomere length, hTERT increases stem cell proliferation, rescues the impaired differentiation, and, in this way, counteracts senescence-induced negative effects [32]. Senescence may be spurred by autophagy deregulation leading to intracellular waste accumulation [33]. Autophagy plays an important role in differentiation and stemness regulation of various cellular types, including MSCs [17]. Its activation has been suggested to counteract bone loss [34] as well as MSC senescence [35]. Autophagy also regulates adipocyte differentiation and lipid accumulation and plays an important role in the balance between white and brown fat [36]. Conversely, impaired autophagy in MSCs can induce metabolic diseases [35, 37–39]. Thus, autophagy is considered an “antiaging dynamic system” because of its contribution to cell renewal and homeostasis [40]. It has been reported that telomerase regulates autophagy [41, 42]; an increase in telomerase activity and autophagy has been demonstrated following calorie restriction [43].

The aim of this study was to understand whether physical activity may promote osteogenic differentiation of CPCs. We investigated the involvement of cellular processes such as autophagy and telomere maintenance in osteogenic differentiation by means of PCR array analysis.

Therefore, in order to better understand the effects of physical activity on M-CPCs, we investigated their commitment as well as their modulation of telomerase-related genes, following the half marathon. We evaluated the effects of half marathon also on the autophagic process, as a supposed mechanism influencing M-CPC differentiation. In addition, we performed *in vitro* experiments supporting our findings.

## 2. Materials and Methods

### 2.1. Subjects

**Figure 1:**
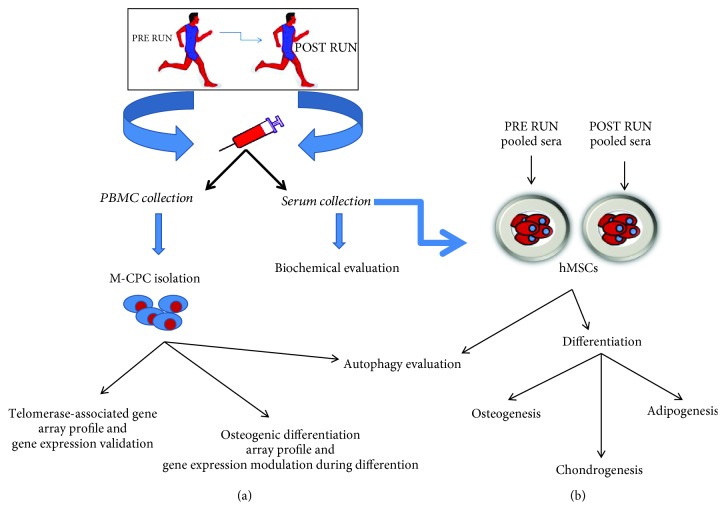
A schematic view describing the work flow and connections between the data obtained by investigations performed *in vivo* (a) and *in vitro* (b).

22 male runners and 10 age-matched male controls were included in the study (Figure 1). Study subjects were healthy and regularly active. Participants were enrolled during sport events called “run for science,” held in Verona (Italy) in April 2016 and April 2017. Twenty-two male runners (median age: 45, 4 ± 11; median BMI: 22.6) carried out a 21.1 km half marathon in 1.40 ± 0.44 hours. Participants were all recreational runners and physically fit, with a median training regimen in the last 10 years: 220 minute/week and with an estimated mean VO_2_ max of 44.9 at the end of the run, classified as great for males with similar mean age [44]. Controls have median age of 44, 7 ± 12, median BMI of 24.2, and estimated VO_2_ of 36-41 [45, 46] and had not been training for 13.4 ± 2 years. All runners and controls underwent a clinical evaluation and a medical history interview in order to exclude comorbidities or drug intake.

Blood was collected from controls and runners at the same time of day. Blood samples, obtained by venipuncture, were collected immediately before and 15 min after the run, respectively. Written informed consent was obtained from all participants, and the study was approved by the ethical committee of Azienda Ospedaliera Universitaria Integrata of Verona, Italy (number 1538; Dec. 3, 2012; local ethical committee of Azienda Ospedaliera Integrata di Verona). The study design and methods comply with the Declaration of Helsinki. Biochemical analyses were performed before and after the run: s-calcium (Ca), s-phosphate (P), carboxy-terminal telopeptide of type I collagen (CTX), ferritin, and chlorine (Cl) were monitored; 25-hydroxy vitamin D (vitD) was also evaluated in order to exclude vitD-deficient individuals.

### 2.2. Serum Collection

In order to evaluate the effects of sera *in vitro*, we collected PRE and POST RUN sera from 10 ml of fresh blood by centrifugation at 400×g. Sera were harvested and frozen in aliquots at −80°C until use. To perform the *in vitro* experiments, individual PRE RUN sera and POST RUN sera, respectively, were pooled.

### 2.3. Circulating Progenitor Cells (CPCs)

To evaluate differentiation ability and modulation of telomerase-related genes, we isolated M-CPCs from heparinized blood as previously reported [11]. In particular, M-CPCs were collected from 50 ml of heparinized blood by a depletion method, performed by two Ficoll procedures in order to remove hematopoietic cells using the antibody cocktail. Initially, peripheral blood mononuclear cells (PBMCs) were isolated by a gradient centrifugation at 800×g for 30 min at 20°C (first Ficoll procedure). Then, to remove the unwanted hematopoietic cells, a RosetteSep antibody cocktail (Stemcell Technologies Inc., Vancouver, Canada) was used with 5 ml of whole blood mixed with the PBMCs obtained by the first Ficoll. The antibody cocktail (against CD3, CD14, CD19, CD38, and CD66b-positive cells), at dilution of 50 *μ*l/ml, was incubated with samples for 20 min at room temperature. Finally, a second Ficoll procedure was performed to remove the unwanted cells linked to antibody cocktail and crosslinked to red blood cells (glycophorin A). Collected cells were then washed in phosphate-buffered saline (PBS). Phenotype analysis was performed as previously described [9].

### 2.4. Analysis of Cell Phenotype

We evaluated the phenotype of M-CPCs by analyzing the gene expression for CD3, CD14, CD19, CD45, CD34, CD73, and CD105 markers, as reported previously [9]. This method allows the analysis of cell phenotype when stringent purification strategies are performed [47].

### 2.5. In Vitro Osteogenic and Adipogenic Differentiation

Sera, obtained from each runner before and after the competition, were mixed in two pools named PRE RUN and POST RUN sera, respectively. In order to study the induction of osteogenic and adipogenic differentiation, respectively, before and after physical exercise, we treated the BM-hMSC cell line (human bone marrow-human mesenchymal stem cells, PromoCell, Heidelberg, Germany) with pooled PRE RUN and POST RUN sera [5].

Pooled sera were added to the above cell line at 10% final concentration. Cells were plated at a density of 5 × 10^4^ cells per well into 24-well plates and cultured up to 21 days. In particular, osteogenic differentiation was performed with osteogenic medium containing osteogenic stimulatory supplements (15%, Stemcell Technologies Inc., Vancouver, Canada), 10^−8^ M dexamethasone, 3.5 mM *β*-glycerophosphate, and 50 *μ*g/ml ascorbic acid (Stemcell Technologies Inc.). The adipogenic differentiation was performed by using 0.5 mM isobutylmethylxanthine, 200 *μ*M indomethacin, 10^−6^ M dexamethasone, and 10 *μ*g/ml insulin in basal medium. Chondrogenic differentiation was performed by culturing hMSCs with mesenchymal stem cell chondrogenic differentiation medium (PromoCell, Heidelberg, Germany). For osteogenic, adipogenic, or chondrogenic differentiation, the medium was changed every 3 days after initial plating.

### 2.6. Total RNA Extraction and Reverse Transcription

Pellets from the differentiated BM-hMSC cell line treated with pooled sera and from CPCs were collected and stored at -80°C. Then, they were processed for RNA extraction with a “RNeasy® protect mini kit” by Qiagen, Hilden, Germany, following the manufacturer's protocol. RNA samples were quantified by a Qubit™ 3 fluorometer using a “Qubit™ RNA HS assay kit” (Invitrogen, Carlsbad, USA). Two micrograms of the extracted RNAs was reverse transcribed with a “first strand cDNA synthesis kit” by GE Healthcare, Little Chalfont, UK, according to the producer's instructions. RNA and cDNA samples were stored at -80°C.

### 2.7. PCR Array

PCR arrays were performed by using TaqMan™ array human osteogenesis (Thermo Fisher Corporation, Waltham, MA, USA, 4414096) and TaqMan™ array human telomere extension by telomerase (Thermo Fisher Corporation, Waltham, MA, USA, 4414187) according to the manufacturer's instructions.

### 2.8. Real-Time RT-PCR

To validate the array profile of M-CPCs as well as to investigate gene expression modulation in M-CPCs and in *in vitro* experiments, we performed real-time PCR analyses as reported previously [10]. Briefly, predesigned, gene-specific primers and probe sets for each gene (CD3, hs00174158_m1; CD14, hs02621496-s1; CD19, hs00174333_m1; CD45, hs00174541_ m1; CD34, hs00156373_m1; CD73, hs00159686_m1; CD105, hs00923996_m1; RUNX2, *hs00231692*_m1; OSTERIX (SP7), hs00541729_m1; COLLAGEN, TYPE I, ALPHA 2 (COL1A2), hs01028956_m1; OSTEONECTIN (SPARC), hs00234160_m1; OSTEOPONTIN (SPP1), hs00167093_m1; BMP1, hs00241807-m1; BMP3, hs00609638-m1; BMP4, hs03676628-s1; BMPR1A, hs1034913-g1; SOX9, hs01107818_m1; COMP, hs004359-m1; MSX1, hs00427183-m1; HNRNPA1, hs01656228-s1; MRE11a, hs00967437-m1; RAD50, hs00990023-m1; TERF1, hs00819517-m1; TERT, hs00972650-m1; COL2A1, hs00264051_m1; PPARG2, hs01115513_m1; B2M, hs999999_m1 (housekeeping); and GAPDH, 0802021 (housekeeping)) were obtained from assay-on-demand gene expression products (Thermo Fisher Corporation, Waltham, MA, USA). For autophagy gene expression, we used the following SYBR green primers: ATG3 FW GGAAGAATATGAAGAGAGTGG RV CTCATCATAGCCAAACAACC, ATG7 FW AGATTGTCCTAAAGCAGTTG RV CCATACATTCACTGAGGTTC, ULK1 FW TCAAAATCCTGAAGGAACTG RV ACCAGGTAGACAGAATTAGC, and *β*-actin FW GATGTATGAAGGCTTTGGTC RV TGTGCACTTTTATTGGTCTC. Ct values for each reaction were determined using TaqMan SDS analysis software (Applied Biosystems; Foster City, California, USA) as reported previously. To calculate relative gene expression levels between different samples, we performed the analyses by using the 2^−*ΔΔ*CT^ method and by using untreated MSC lines as calibrator.

### 2.9. Immunofluorescence

Immunofluorescence analyses were performed to investigate osteogenic and adipogenic differentiation or autophagy in *in vitro* experiments. After 21-day culture, cells were fixed and processed according to the manufacturer's protocols. Primary antibodies were diluted (as reported in the datasheet) in antibody dilution buffer and incubated overnight at 4°C. To evaluate osteogenic differentiation and autophagy, we used osteocalcin C8 antibody (sc74495, Santa Cruz, Dallas, Texas, USA) and LC3B (cat. #2775, Cell Signaling, Leiden, The Netherlands), respectively. Assessment of adipogenic stimuli was achieved by testing perilipin D1D8 (cat. #12589, Cell Signaling). Slides were then incubated with secondary antibodies Alexa Fluor® 594 anti-rabbit (cat. #r37117), Alexa Fluor® 488 anti-rabbit (cat. #a-11034), and goat mouse fluorescein conjugated (cat. Ap124f, Millipore, Burlington, Massachusetts, USA). Nuclear staining was performed by ProLong™ Gold Antifade Mountant with DAPI. Images were recorded using a Leica (Wetzlar, Germany) TCS SP5 AOBS inverted confocal microscope at 63x. To express data in a semiquantitative way, four different fields were measured for each sample, in three independent experiments with about 80–100 total cells.

### 2.10. Western Blotting

To investigate protein levels related to osteogenic, chondrogenic, and adipogenic differentiation or to autophagy in *in vitro* experiments, we performed Western blot analyses. Protein extraction was performed using RIPA buffer (Thermo Fisher Scientific, Waltham, MA, USA) according to the manufacturer's protocol. A BCA assay (Thermo Fisher Scientific, Waltham, MA, USA) was used to determine protein concentrations. Protein samples were diluted in 4x Laemmli's sample buffer (Bio-Rad, CA, US), heated for 5 min at 95°C, and separated by sodium dodecyl sulfate-polyacrylamide gel electrophoresis (SDS PAGE), using a mini-PROTEAN® TGX™ Precast gradient 4-20% gel (Bio-Rad, CA, US), followed by transfer onto polyvinylidene difluoride (PVDF) membranes (Thermo Fisher Scientific, Waltham, MA, USA). PVDF membranes were probed with the primary and secondary antibodies reported in Table 1. Signals were detected using a chemiluminescence reagent (ECL, Millipore, Burlington, MA, USA) according to the manufacturer's instructions. Images were acquired by a LAS4000 Digital Image Scanning System (GE Healthcare, Little Chalfont, UK). Densitometric analysis was performed by using ImageQuant software (GE Healthcare, Little Chalfont, UK), and the relative protein band intensity was normalized to *β*-actin.

### 2.11. Alkaline Phosphatase Activity

To evaluate the activity of alkaline phosphatase during osteogenic differentiation *in vitro*, MSCs were cultured in the presence of either PRE RUN or POST RUN sera. After 14 days of culture, cytoplasmic ALP was analyzed by using the Alkaline Phosphatase Kit 86-C (Sigma Aldrich GmbH, Munich, Germany) as previously reported [48]. ALP activity was calculated by multiplying the number of positive cells × the staining intensity value (from 0 to 4) in 100 total cells according to the manufacturer's instructions.

### 2.12. Alizarin Red Staining

To evaluate calcium deposition during osteogenic differentiation *in vitro*, we performed Alizarin red staining as previously described [8]. Briefly, after 21 days of culture in the presence of osteogenic medium, cells were fixed with 70% ethanol, washed three times with water, and stained for 5 min with 40 mM Alizarin red S at pH 4.1. Cells were then gently rinsed with 1x phosphate-buffered saline for 15 min. The area stained with Alizarin red S was quantified in three independent experiments by using ImageJ software (NIH, Bethesda, MD, USA) as previously reported [49].

### 2.13. Oil Red O Staining

To evaluate the formation of lipid droplets during adipogenic differentiation *in vitro*, we performed Oil red O staining. The cells, cultured in adipogenic medium with added PRE RUN and POST RUN sera, respectively, were stained with Oil red O according to the manufacturer's instructions. The total area of red pixels in the Oil red O-stained droplets/cell was determined by using the ImageJ analysis as previously reported [50]. In particular, means ± SD of red stained areas, measured at 40x magnification, were calculated in three different fields/slides and expressed as the percentage compared to the total area.

### 2.14. Alcian Blue Staining

To evaluate chondrogenic differentiation in *in vitro* experiments, we performed Alcian blue staining after 21 days of culture as previously reported [12]. Briefly, the cells cultured in chondrogenic differentiation medium and with PRE or POST RUN sera were fixed with 95% methanol and then stained with 1% Alcian blue 8GX HCl overnight. Then, cell slides were washed and observed under a microscope.

### 2.15. Statistical Analysis

Results were expressed as the mean ± SD. Statistical analysis was assessed by Student's paired *t*-test. Differences with *p* < 0, 05 were considered significant. We performed also correlation analyses by Pearson's test to measure linear dependence between the expression of a single autophagy gene and the transcription factor RUNX2 or SOX9. For in vitro data, analyses were applied to experiments carried out at least three times. Statistical analyses were performed using SPSS for Windows, version 22.0 (SPSS Inc., Chicago, IL, USA).

## 3. Results

### 3.1. Biochemical Data

Results of biochemical evaluations in sera collected before (PRE RUN) and after the run (POST RUN) are reported in Table 2. None of the subjects showed hypovitaminosis D at the baseline. Electrolytes, in particular, sodium and potassium, showed significant variations due to dehydration, as well as iron serum concentration. Ferritin levels were also increased in POST RUN sera (*p* < 0.05).

### 3.2. Physical Exercise Promotes Mesenchymal Stem Cell Differentiation

We evaluated the progenitor cells' phenotype as previously reported [12]. In Table 3, we reported the expression of cluster differentiation (CD) of mesenchymal cell phenotype (CD 105 and CD 73). In order to prove the low hematopoietic contamination after depletion, we reported also values of hematopoietic cell phenotype CDs. No phenotypical difference was observed between PRE RUN- and POST RUN-selected cells. In order to evaluate if physical exercise induces mesenchymal differentiation in circulating progenitors, we analyzed the differentiation profiles in PRE RUN and POST RUN CPCs by the osteogenesis array and subsequent RT real-time PCR.

**Figure 2:**
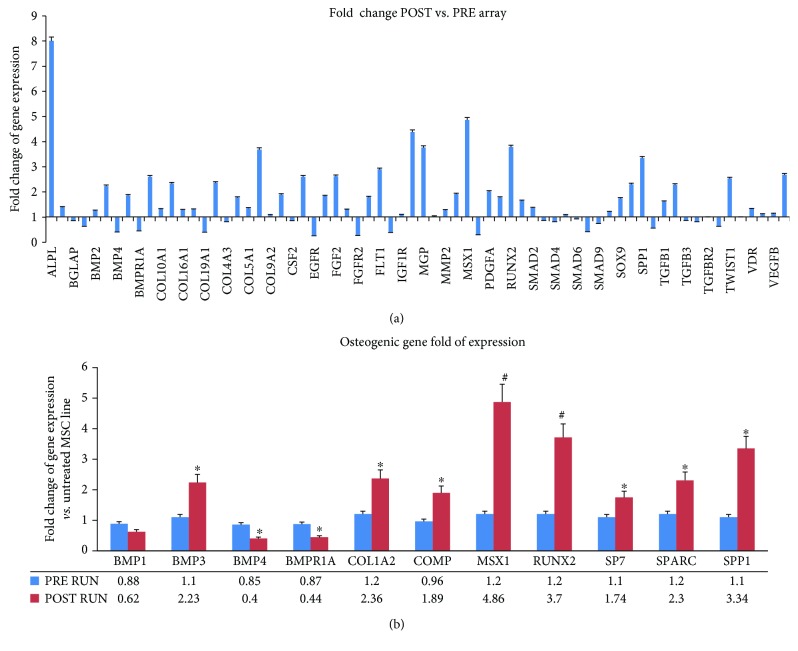
Expression levels plotted for 68 genes evaluated by TaqMan™ Human Osteogenesis Array in M-CPCs (a). Real-time PCR single assay validation of BMP1, BMP3, BMP4, BMPR1A, COL1A2, COMP, MSX1, RUNX2, SOX9, SPARC, and SSP1 was performed in M-CPCs of runners (b). Fold change of gene expression is reported as normalized 2^−*ΔΔ*CT^ values. ^∗^*p* < 0.05; ^#^*p* < 0.01. In blue: PRE RUN; in red: POST RUN.

The fold changes of gene expression (Figure 2(a)) obtained by the osteogenesis array are listed in Supplemental [Supplementary-material supplementary-material-1].

In order to validate these findings, we performed RT real-time PCR assays for several genes. We investigated the expression of SP7, the RUNX2 downstream gene, as well.

Most of the investigated genes were expressed at higher levels in POST RUN M-CPCs compared to PRE RUN M-CPCs (Figure 2(b)). The higher expression levels indicated a strong commitment of M-CPCs to osteogenic differentiation. In particular, RUNX2 expression in POST RUN was >3-fold higher compared to PRE RUN CPCs (*p* < 0.01); RUNX2 downstream genes COL1A2 (*p* < 0.05), SPARC (*p* < 0.05), SP7 (*p* < 0.05), and SPP1 (*p* < 0.01) in turn confirmed the osteogenic trend, as their expression was higher too.

However, we observed lower POST RUN expression levels of BMP4 (*p* < 0.05) and BMPR1a (*p* < 0.05), compared to PRE RUN levels. Ten controls were also included in the study, in order to monitor any bias due to technical procedures. In control samples obtained at time 0 and after 2 hrs, no differences were observed in gene expression evaluated by RT real-time PCR ([Supplementary-material supplementary-material-1]).

**Figure 3:**
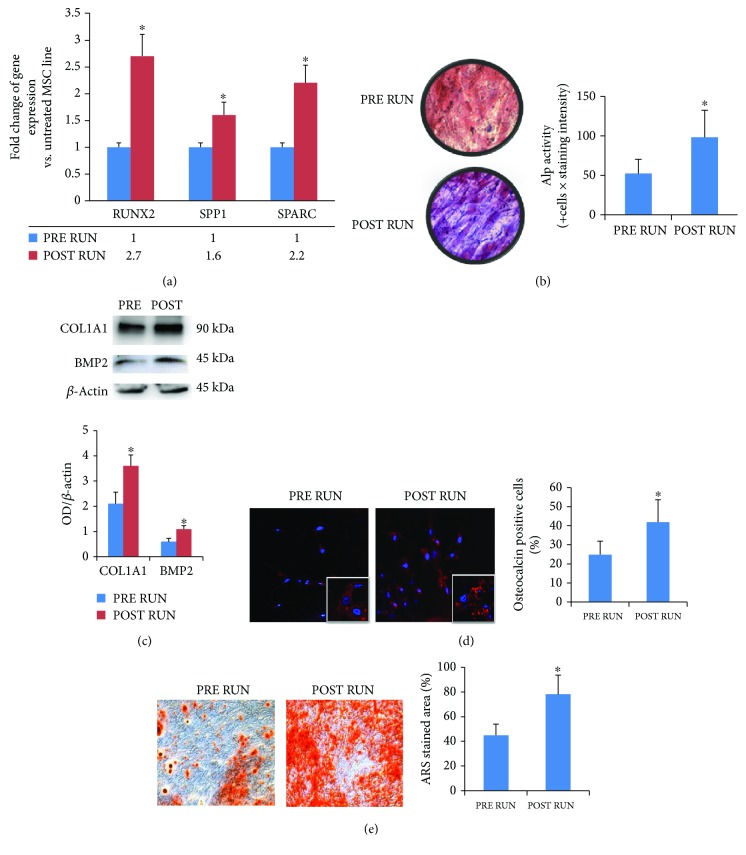
Effects of sera on a mesenchymal stem cell line. Gene expression profiles confirmed the osteogenic commitment observed in M-CPCs (a); ALP activity (b) and COL1A1 and BMP2 protein expression (c) as well as osteocalcin-positive cells (10x, inset 60x) (d); calcium deposition, evaluated by Alizarin red staining (10x) (e), indicated the increased osteogenic maturation of cells treated with POST RUN sera. The *in vitro* analyses were performed in three independent experiments, ^∗^*p* < 0.05.

Considering that the observed modulation in CPCs might be induced by circulating factors, we then tested the effects of sera in *in vitro* experiments. We analyzed the effects of PRE RUN and POST RUN sera on a hBM-MSC line. Findings from the MSC line culture supplemented with runners' sera confirmed the data obtained in M-CPCs. In detail, gene expression profiles (after 7 days of culture for RUNX2 and after 14 days of culture for SPP1 and SPARC) (Figure 3(a)), alkaline phosphatase activity (Figure 3(b)), COL1A1 and BMP2 protein expression (Figure 3(c)), osteocalcin expression (Figure 3(d)), and calcium deposition (evaluated by Alizarin red staining) (Figure 3(e)) indicated the increased osteogenic differentiation and maturation of cells treated with POST-RUN sera.

**Figure 4:**
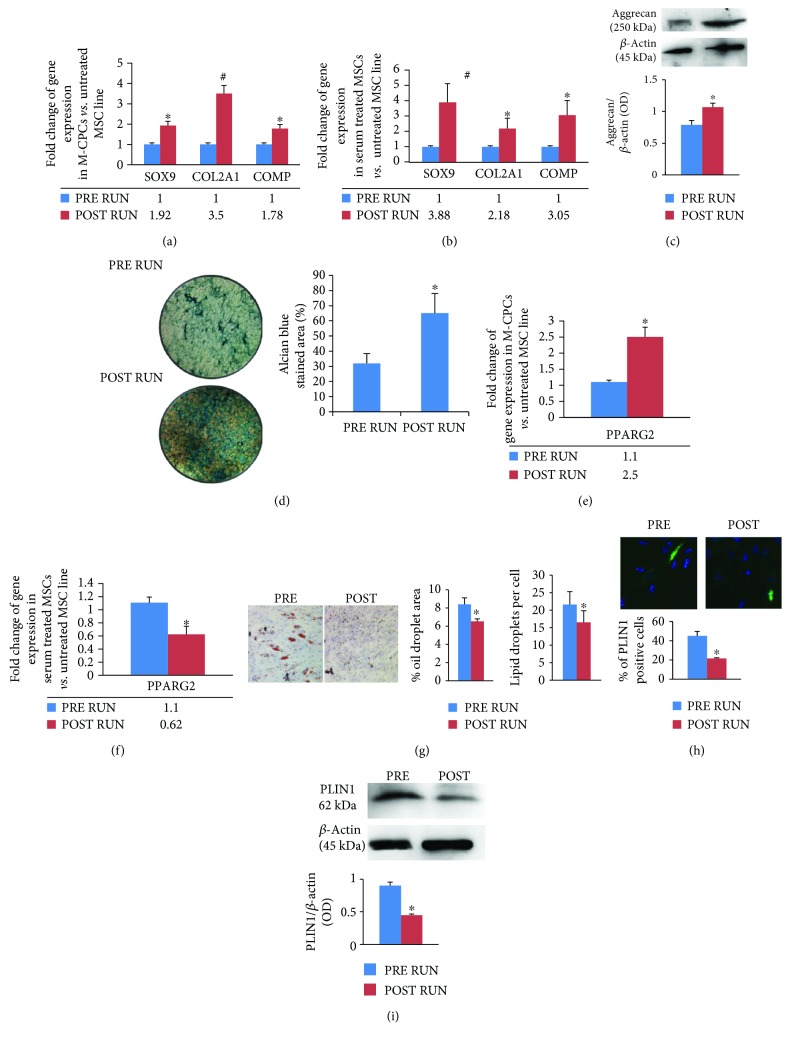
Physical exercise improves chondrogenic differentiation and affects adipogenic differentiation. Besides SOX9 upregulation, we observed also a higher expression of COL2A1 and COMP in M-CPCs after the run (a). *In vitro* experiments showed an increased chondrogenic differentiation in the MSC line cultured in the presence of POST RUN sera. In fact, a higher expression of chondrogenic genes (b) and of Aggrecan protein (c) as well as more intense Alcian blue staining ((d); 10x) was observed. Adipogenic differentiation. PPARG2 gene expression was higher in POST RUN M-CPCs (e). Conversely, the MSC line treated with POST RUN sera showed reduced adipogenic differentiation. In fact, PPARG2 gene expression levels were lower (f); the number of lipid droplets ((g); 10x) and of PLIN1-positive cells ((h); 40x) as well as PLIN1 protein levels (i) was reduced in the MSC line treated with POST RUN sera. The *in vitro* analyses were performed in three independent experiments. ^∗^*p* < 0.05; ^#^*p* < 0.01.

To investigate the chondrogenic commitment in M-CPCs, we analyzed the expression of SOX9 transcription factor and of the cartilage-specific COL2A1 and COMP genes by RT real-time PCR. In particular, the higher expression of COL2A1 (*p* < 0.01) as well as of COMP (*p* < 0.05) in POST RUN M-CPCs confirmed the clues obtained from the chondrogenic determinant SOX9 (*p* < 0.05) (Figure 4(a)). The hBM-MSC line supplemented with runners' sera confirmed the enhanced chondrogenic differentiation when cells were cultured in the presence of POST RUN sera. The increased expression of chondrogenic genes (SOX9 after 7 days of culture; COL2A1 and COMP after 14 days of culture) (Figure 4(b)) of Aggrecan protein (Figure 4(c)) as well as stronger Alcian blue staining (Figure 4(d)) was detected in cultures supplemented with POST RUN sera.

To evaluate M-CPC adipogenic commitment, we analyzed the expression of the adipogenic transcription factor PPARG2. Data obtained by RT real-time PCR showed that the PPARG2 gene expression was higher in POST RUN M-CPCs (Figure 4(e)) (*p* < 0.05). No variations were observed in the expression of the above genes in progenitor cells from controls ([Supplementary-material supplementary-material-1]). However, when we treated the hBM-MSC line with POST RUN sera, PPARG2 gene expression levels (after 7 days of culture) were lower compared to those observed with PRE RUN serum treatment (Figure 4(f)) (*p* < 0.05). These latter data were confirmed by Oil red O staining of cells after 14 days of culture: a reduction of lipid droplets (Figure 4(g)) and a decreased number of PLIN1-positive cells (Figure 4(h)) as well as lower PLIN1 protein levels (Figure 4(i)) were observed.

We did not observe any effects related to osteogenic, adipogenic, or chondrogenic differentiation in the hBM-MSC line treated with control sera ([Supplementary-material supplementary-material-1]).

### 3.3. Physical Exercise Affects the Expression of Telomerase-Associated Genes and Enhances Autophagy

**Figure 5:**
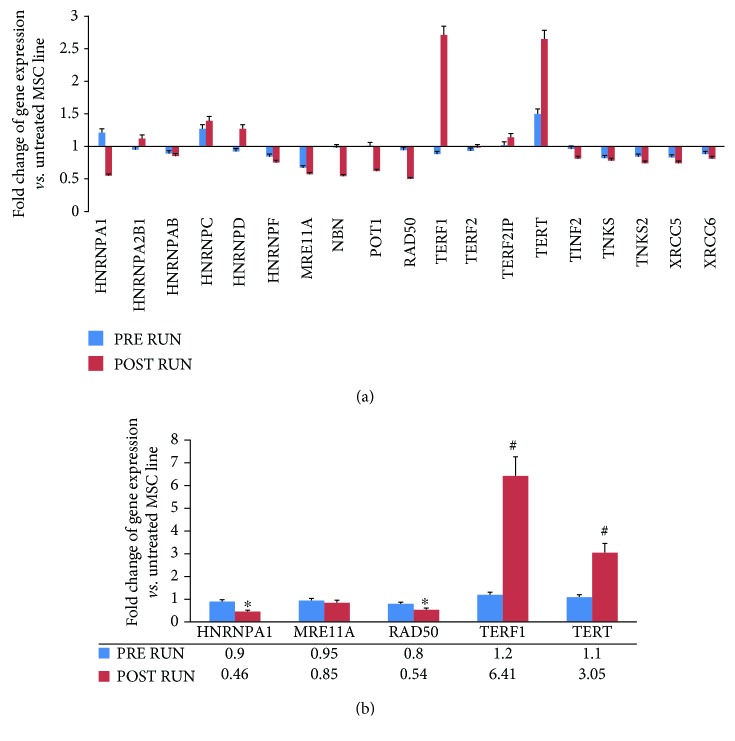
Fold change of gene expression plotted for 19 genes evaluated by TaqMan™ Human Telomerase Array in pooled M-CPCs of runners (a). In M-CPCs of runners, we performed real-time PCR single assay validations of HNRNPA1, MRE11A, RAD50, TERF1, and TERT (b). Fold change of gene expressions is reported as normalized 2^−*ΔΔ*CT^ values. ^∗^*p* < 0.05; ^#^*p* < 0.01.

We analyzed the expression of telomerase-associated genes and autophagy-related genes in order to evaluate the involvement of these cellular processes in the differentiation of circulating progenitors following physical exercise. Telomerase-associated genes, evaluated by gene array analysis in M-CPCs, were differentially expressed after physical exercise (*p* < 0.01) (Figure 5(a)). Supplemental [Supplementary-material supplementary-material-1] reports in detail POST-RUN fold changes of gene expression.

Thereafter, we performed RT real-time PCR assays to validate the array data, which were confirmed as shown in Figure 5(b). Notably, both TERT and TERF1 POST RUN expression levels were higher than PRE RUN levels (*p* < 0.01), while POST RUN expression levels of several DNA repair genes (e.g., RAD50 and HNRNPA1) were lower (*p* < 0.05). No differences were observed in controls ([Supplementary-material supplementary-material-1]).

**Figure 6:**
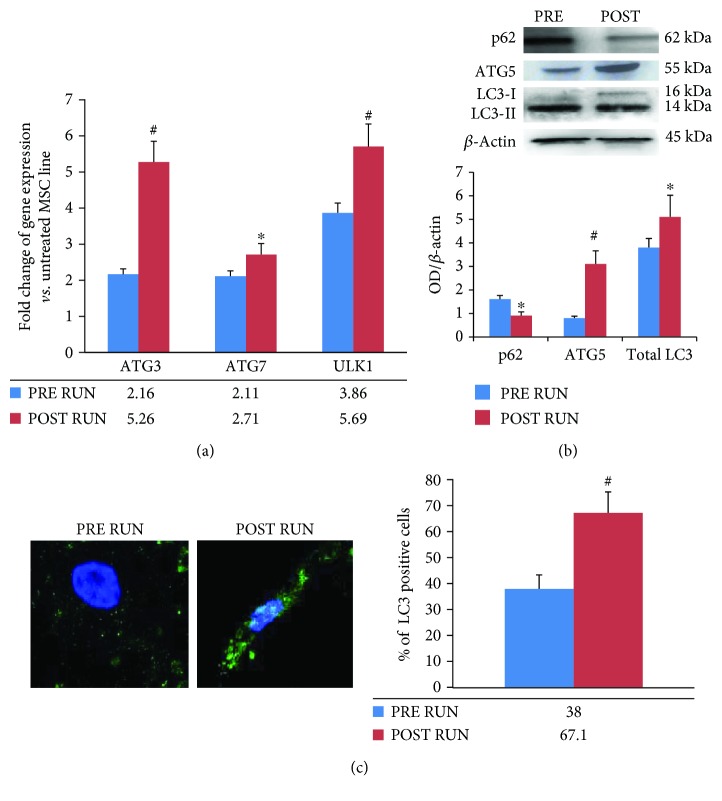
Physical exercise induced the upregulation of autophagy-related genes in M-CPC (a). To confirm this finding, we treated a MSC line with PRE RUN and POST RUN sera and we analyzed p62, ATG5, and LC3 expression. As shown in (b), MSCs treated with POST RUN sera expressed lower p62 and higher ATG5 and total LC3 (both LC3I and LC3II) protein levels compared to cells treated with PRE RUN sera. This last finding was confirmed by analyzing the number of cells expressing LC3 (c), evaluated by a fluorescence confocal microscope, 63x. *In vitro* analyses were performed in three independent experiments, ^∗^*p* < 0.05; ^#^*p* < 0.01.

Finally, we investigated the expression of autophagy-related genes, in order to gain information about the physical exercise-induced modulation of this vital cellular process. We analyzed ATG3, ATG7, and ULK1, in PRE and POST RUN M-CPCs. Autophagy-related genes appeared to be upregulated in POST RUN M-CPCs (Figure 6(a)) whereas no differences were found in controls ([Supplementary-material supplementary-material-1]).

To confirm these findings, we treated a hBM-MSC line with PRE and POST RUN sera. We found that cells treated with POST RUN sera expressed lower levels of the autophagic flux marker p62 and higher ATG5 and LC3 levels, respectively, compared to cells treated with PRE RUN sera (Figures 6(b) and 6(c)).

Interestingly, by using Pearson's correlation test, we found a positive correlation between ATG3 and SOX9 expression (*r* = 0.95) and ATG3 and RUNX2 expression (*r* = 0.98). Similarly, positive correlations between ULK1 and SOX9 expression (*r* = 0.96) and ULK1 and RUNX2 expression (*r* = 0.97) were observed.

## 4. Discussion

Our findings call for some comments on the biological effects of half marathon. Biochemical evaluations showed a significant increase of iron levels, possibly related to tissue injury, as previously suggested [51], while the increased ferritin levels might be attributed to the inflammatory process related to the run. However, the increased serum ferritin levels point also towards the autophagic process activation in response to physical activity. In fact, it has been demonstrated that serum ferritin is secreted from macrophages by an “atypical” vesicular system involving the autophagic process [52]. In particular, ferritin gets into the endolysosomal system via NCOA4 autophagy receptor [53]. Therefore, autophagy is involved in the regulation of cellular redox status modulated by physical activity [54], through the degradation of iron-binding proteins such as ferritin [55].

In this study, we also confirmed the role of physical exercise in inducing osteogenic differentiation. In particular, for the first time, we reported osteogenic gene expression modulation during physical activity. The expression of most osteogenesis-related genes, namely, RUNX2, MSX1, and SPP1, appeared upregulated after running. Array data showed increased levels of BMP2 and BMP6, according to their role in promoting osteogenesis [56]. The *in vitro* effects of sera on the hBM-MSC line confirmed the enhanced osteogenic differentiation due to physical exercise. On the other hand, the observed lower expression of BMP4 in M-CPCs was intriguing: it might be explained considering that BMP4 plays a dual role both in adipogenesis and in metabolism [57]. Consistently, we observed a downregulation of BMPR1a gene in POST RUN M-CPCs, as BMP4 signal transduction is mediated by BMPR1a receptor [58]. In addition, it has been demonstrated that BMP4 reduces PPARG2 and increases PPARG1 expression in order to drive the terminal differentiation to white-like adipocyte at brown adipocyte's expense [59]. Indeed, we observed an increased expression of PPARG2 in M-CPCs. This finding is in agreement with the role of PPARG2 in regulating insulin sensitivity and glucose utilization as well as energy homeostasis [60]. Conversely, we did not observe increased PPARG2 gene expression in the hBM-MSC line treated with POST RUN sera. In addition, the number of lipid droplets as well as PLIN1 protein levels was reduced in MSCs treated with POST RUN sera suggesting that physical activity modulates negatively adipogenic differentiation. Moreover, we observed a higher expression of BMP3 after the athletic performance. Unlike BMP2 and BMP6, BMP3 reduces osteogenesis; it also promotes preadipocyte proliferation by activating the TGFbeta/ACTIVIN-signaling pathway. Interestingly, we have previously demonstrated that physical exercise modulates miRNA expression in progenitor cells promoting osteogenesis [61]. Otherwise, by acting on WISP1, BMP3 stimulates MSC proliferation [62]. This finding explains our results related to the expression of both TERT and TERF1 genes which was higher in M-CPCs after the run. We assume that physical exercise may exert a dual action on progenitor cells by enhancing their proliferation and by inducing their commitment and differentiation. It is worth mentioning that hTERT plays an important role among the cellular processes affecting osteogenic differentiation [31, 32]. Murine MSCs with knock-down telomerase activity cannot differentiate into adipocytes or chondrocytes [63] while telomerase-overexpressing cells have an increased osteogenic ability [32]. However, TERT expression in MSCs is debatable. It is known that telomerase activity is reduced in differentiating progenitor cells with reduced proliferative ability [30]. Telomerase levels in MSCs are low or absent [64] and can be upregulated by stimuli [30]. Interestingly, among the stimuli inducing telomerase expression, oxidative stress plays an important role [65]. Therefore, the increased telomerase expression observed in POST RUN CPCs could be due to the oxidative stress following physical activity.

In addition, in POST RUN M-CPCs, we observed the upregulation of genes associated with chondrogenic differentiation such as SOX9, COMP, and COL2A1. The *in vitro* experiments confirmed these findings. These data not only demonstrate that physical exercise induces progenitor cell commitment to the chondrogenic lineage but also suggest that chondrocytic maturation is accomplished. We cannot exclude that the PRE RUN and POST RUN changes observed in gene expression result from the recruitment of different cell types. However, it has been reported that the amount of M-CPCs is not influenced by physical activity [6], at least immediately after the athletic performance. In fact, it has been reported that bone marrow MSC number increases following physical activity [21]. Therefore, we can speculate that an extension of the observation interval might disclose an increased number of M-CPCs.

In M-CPCs, we observed an increased expression of genes related to autophagy, an important process involved in osteogenic differentiation [66]. These data were confirmed *in vitro*. In fact, positive LC3 cells and total LC3 (LC3I+LC3II) as well as ATG5 protein expression increased while p62 decreased in MSCs treated with POST RUN sera. This finding is in agreement with our previous study where we observed, by proteomic analysis of runners' sera, the overexpression of Von Willebrand factor (VWF) after physical exercise [5]. Indeed, it has been demonstrated that autophagy regulates endothelial cell secretion of VWF [67]. This evidence supports the observed elevated ferritin levels in sera after physical activity; we assume that a strong induction of autophagy may occur during an endurance trial in various cell types. Moreover, we observed a positive correlation between ATG3 and ULK1 gene expression and SOX 9 and RUNX2 gene expression in circulating progenitors. The above observations highlight the positive effect of autophagy on the chondrogenic and osteogenic differentiation, respectively; moreover, we assume that autophagy was enhanced by physical exercise as a protective mechanism in response to oxidative stress. Thus, induced autophagy promoted progenitors' chondrogenic and osteogenic differentiation. Interestingly, it has been demonstrated that telomerase regulates the autophagic process through a glycolysis enzyme (HK2) involved in the catabolism from glucose to glucose-6-phosphate [41].

In summary, we suppose that the increased expression of chondrogenic and osteogenic genes in M-CPCs following exercise is due to enhanced autophagy. On the other hand, PPARG2 gene-increased expression is a consequence of insulin signaling modulation that occurs during physical activity.

Interestingly, besides the upregulation of autophagy and telomerase-associated genes, we observed downregulation of several DNA repair-related genes. This is a peculiar result since it is known that autophagy may induce either cell survival or apoptosis on the basis of the genomic damage extent [68]. A reduced availability of proteins involved in DNA repair induces apoptosis [69]. It is known that stem cells respond to DNA damage by activating the DNA repair systems [70, 71]. DNA repair is important for cell survival, while it may lead as well to the accumulation of genomic alterations in stem cells. Moreover, it has been shown that DNA repair activity changes from undifferentiated to differentiated hMSCs [72]. In fact, undifferentiated adult hMSCs have higher repair activity and are resistant to radiation, while predifferentiated hMSCs may be sensitive to radiation and undergo apoptosis.

Considering this complex background, we conclude that physical exercise, e.g., half marathon, promotes autophagy in progenitor cells in response to oxidative stress, along with the differentiation process. Indeed, the upregulated TERT and TERF1 gene expression suggests active recruitment of MSCs and enhanced differentiation ability. The observed downregulation of DNA repair genes can be related to autophagy as well [68]. Consequently, predifferentiated cells harboring genome alterations might be eliminated, promoting enhanced differentiation of progenitors after physical activity.

So far, we can conclude that half marathon physical commitment promotes osteogenic and chondrogenic differentiation as well as autophagy, through a complex biological interplay. Autophagy modulation in MSCs appears of great interest for new therapeutic approaches. In fact, autophagy in MSCs enhances their proangiogenic and immunomodulatory characteristics hence highlighting their therapeutic potential against several diseases [73]. In this context, our findings disclose novel insights into the benefits of physical exercise in healthy and chronic degenerative conditions.

## Figures and Tables

**Table 1:** Antibody list.

Antibody	Ab dilution	Origin	Secondary antibody
ATG5 (D5F5U)	1 : 1000	Cell Signaling, 12994	Anti-rabbit (Cell Signaling, 7074)
LC3B	1 : 1000	Cell Signaling, 2775	Anti-rabbit (Cell Signaling, 7074)
Aggrecan (BC-3)	1 : 1000	Thermo Fisher Scientific	Anti-mouse (Cell Signaling, 7076)
PLIN1 (D1D8)	1 : 1000	Cell Signaling, 12589	Anti-rabbit (Cell Signaling, 7074)
BMP2	1 : 1000	Proteintech, MCR, UK	Anti-mouse (Cell Signaling, 7076)
COL1A1 (3G3)	1 : 200	Santa Cruz Biotech., Dallas, TX, USA	Anti-mouse (Cell Signaling, 7076)
SQSTM1/P62	1 : 1000	Cell Signaling, 5114	Anti-rabbit (Cell Signaling, 7074)
*β*-actin (BA3R)	1 : 5000	Thermo Fisher Scientific, Waltham, MA, USA	Anti-mouse (Cell Signaling, 7076)

**Table 2:** Biochemical data of the study population (22 runners).

Parameter	PRE RUN (baseline values) (mean ± SD)	POST RUN values (mean ± SD)	*p* value
Age (yrs)	45.4 ± 11		
Fe (mcg/dl)	15.15 ± 5.6	21.7 ± 6.39	*p* < 0.05
Mg (mg/dl)	0.84 ± 0.04	0.72 ± 0.05	*p* < 0.005
P (mg/dl)	1.12 ± 0.2	1 ± 0.2	NS
Ca (mg/dl)	2.4 ± 0.08	2.48 ± 0.11	NS
Na (mEq/l)	141 ± 0.87	144 ± 1.68	*p* < 0.001
K (mEq/l)	4.5 ± 0.27	4.84 ± 0.33	*p* < 0.05
Cl (mEq/l)	103 ± 1.13	103.8 ± 2.48	NS
Ferritin (ng/ml)	173 ± 170	198.7 ± 194	*p* < 0.05
CTx (ng/ml)	−0.3 ± 0.128	0.36 ± 0.09	NS
25-OH vitamin D (ng/ml)	52.7 ± 10.18	—	—

**Table 3:** Cell phenotype of cells harvested from 22 runners.

Cluster differentiation transcript	PRE RUN (%)	POST RUN (%)	*p* value
CD105	66 ± 0.5	68 ± 0.6	NS
CD73	72 ± 0.3	71 ± 0.4	NS
CD3	0	0	NS
CD14	0.4 ± 0.05	0.5 ± 0.06	NS
CD19	0	0	NS
CD45	1.8 ± 0.4	1.9 ± 0.5	NS
CD34	Low level	Low level	NS

## Data Availability

The biochemical data, array data, gene expression data, Western blot data, and staining data used to support the findings of this study are included within the article. The gene expression data of controls used to support the findings of this study and the data related to array analyses are included within the supplementary information file.

## Supplementary Materials

Supplementary Materials Supplemental Table 1: osteogenic Array analyses. Supplemental Table 2: telomerase related genes analyses. Figure 1S: RT real-time PCR data: expression of osteogenic genes in control samples obtained at time 0 and after 2 hrs. Figure 2S: RT real-time PCR data: expression of adipogenic genes in control samples obtained at time 0 and after 2 hrs. Figure 3S: expression of osteogenic (RUNX2), adipogenic (PPARG2), or chondrogenic (SOX9) transcription factors (A) and Alizarin red staining (B) in the MSC line treated with control sera. Figure 4S: RT real-time PCR data: expression of telomerase-related genes in control samples obtained at time 0 and after 2 hrs. Figure 5S: RT real-time PCR data: expression of autophagy-related genes in control samples obtained at time 0 and after 2 hrs.
