# Supplementary material for: Increased Gene Expression of RUNX2 and SOX9 in Mesenchymal Circulating Progenitors Is Associated with Autophagy during Physical Activity
Source: Oxid Med Cell Longev. 2019 Oct 15;2019:8426259. doi: 10.1155/2019/8426259 (PMC6815530; doi:10.1155/2019/8426259)
Supplement: Supplementary Materials — Supplemental Table 1: osteogenic Array analyses. Supplemental Table 2: telomerase related genes analyses. Figure 1S: RT real-time PCR data: expression of osteogenic genes in control samples obtained at time 0 and after 2 hrs. Figure 2S: RT real-time PCR data: expression of adipogenic genes in control samples obtained at time 0 and after 2 hrs. Figure 3S: expression of osteogenic (RUNX2), adipogenic (PPARG2), or chondrogenic (SOX9) transcription factors (A) and Alizarin red staining (B) in the MSC line treated with control sera. Figure 4S: RT real-time PCR data: expression of telomerase-related genes in control samples obtained at time 0 and after 2 hrs. Figure 5S: RT real-time PCR data: expression of autophagy-related genes in control samples obtained at time 0 and after 2 hrs. [file 8426259.f1.zip › mat.8426259.v3.pptx]

## Slide 1
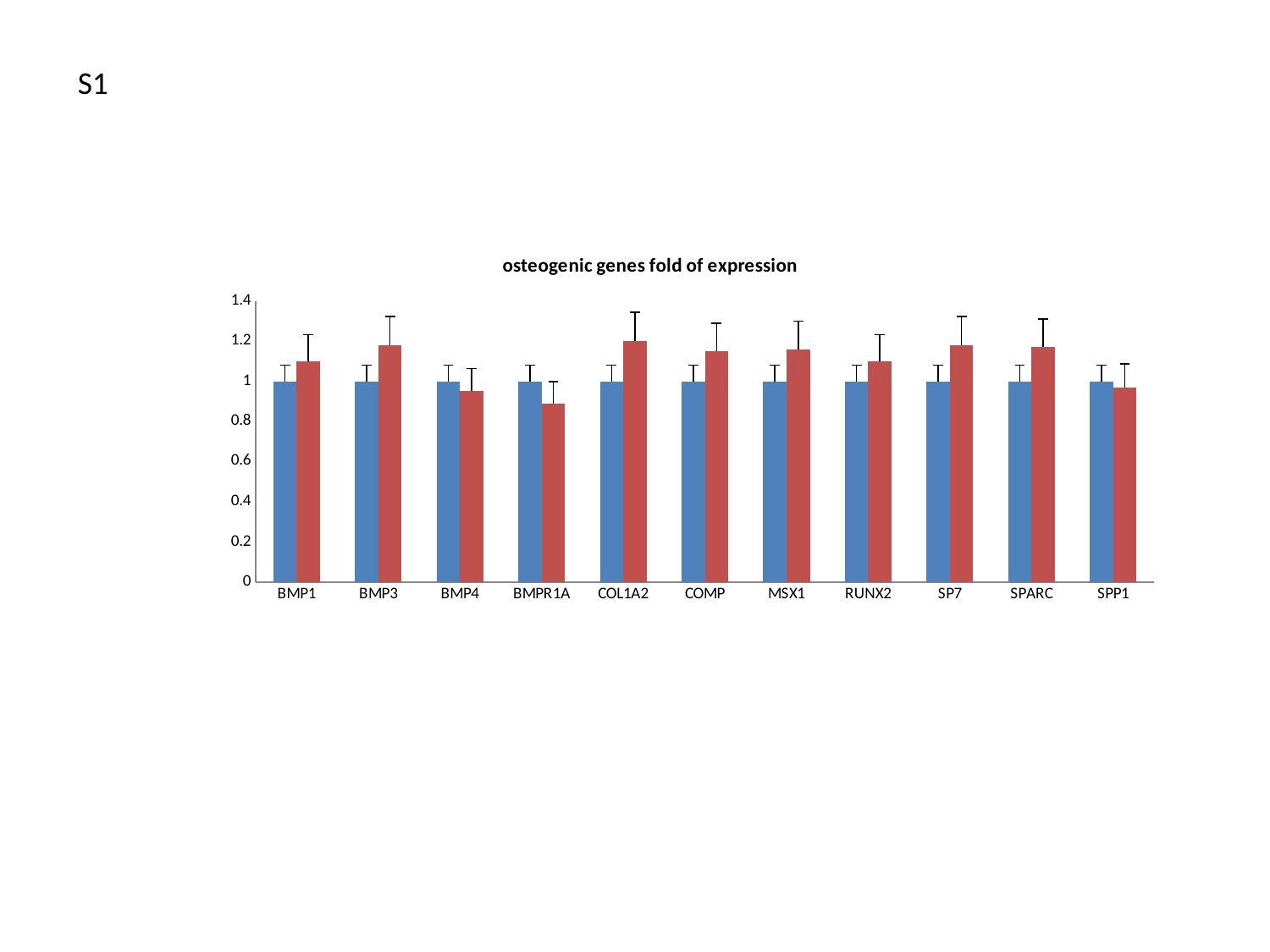

S1
### Chart: osteogenic genes fold of expression
| Category | Controls PRE | Controls POST |
|---|---|---|
| BMP1 | 1.0 | 1.1 |
| BMP3 | 1.0 | 1.1800000000000008 |
| BMP4 | 1.0 | 0.9500000000000004 |
| BMPR1A | 1.0 | 0.8900000000000002 |
| COL1A2 | 1.0 | 1.2 |
| COMP | 1.0 | 1.149999999999999 |
| MSX1 | 1.0 | 1.159999999999999 |
| RUNX2 | 1.0 | 1.1 |
| SP7 | 1.0 | 1.1800000000000008 |
| SPARC | 1.0 | 1.1700000000000008 |
| SPP1 | 1.0 | 0.9700000000000002 |

## Slide 2
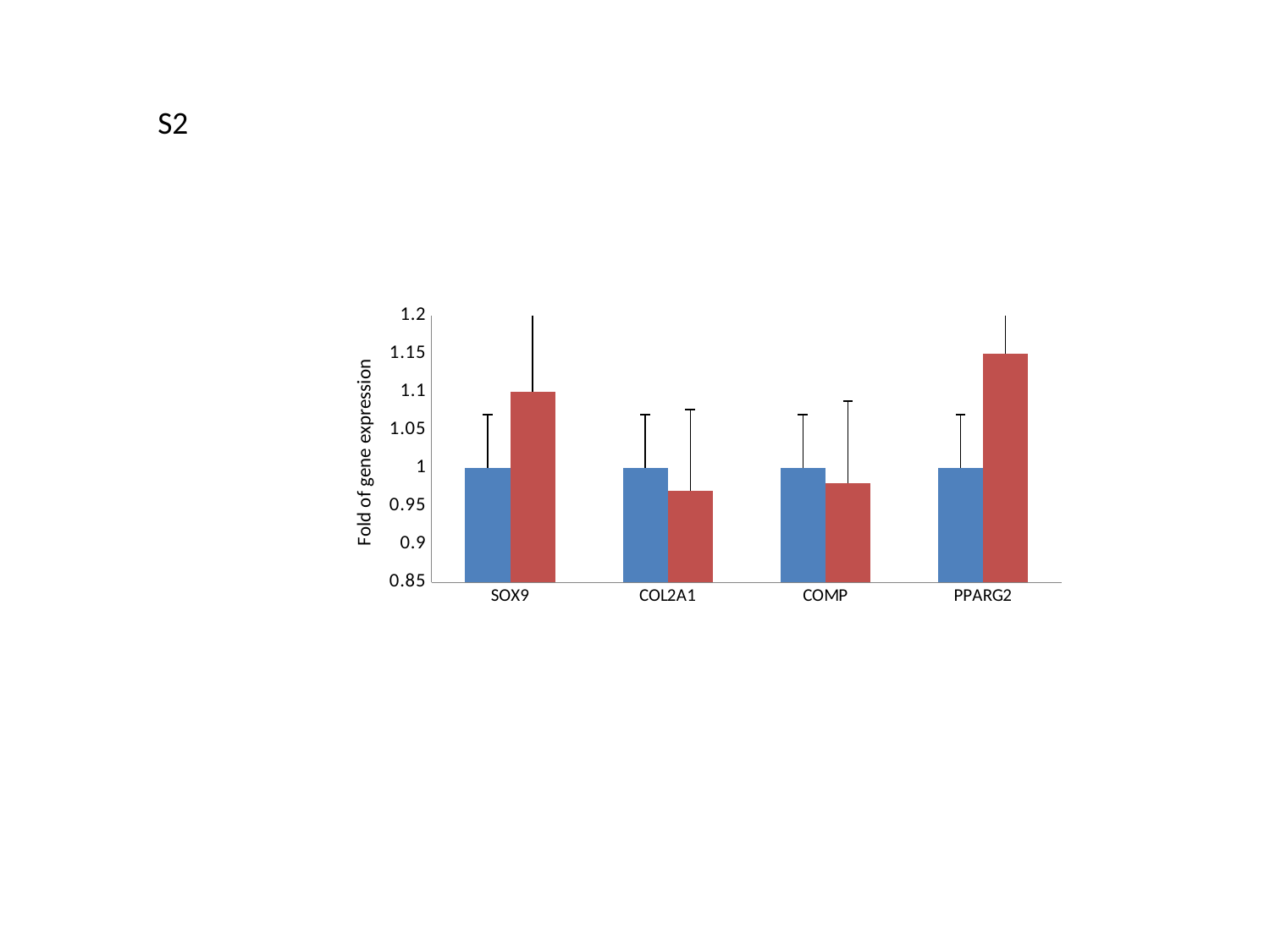

S2
### Chart
| Category | CONTROLS PRE | CONTROLS POST |
|---|---|---|
| SOX9 | 1.0 | 1.1 |
| COL2A1 | 1.0 | 0.9700000000000003 |
| COMP | 1.0 | 0.98 |
| PPARG2 | 1.0 | 1.149999999999999 |Fold of gene expression

## Slide 3
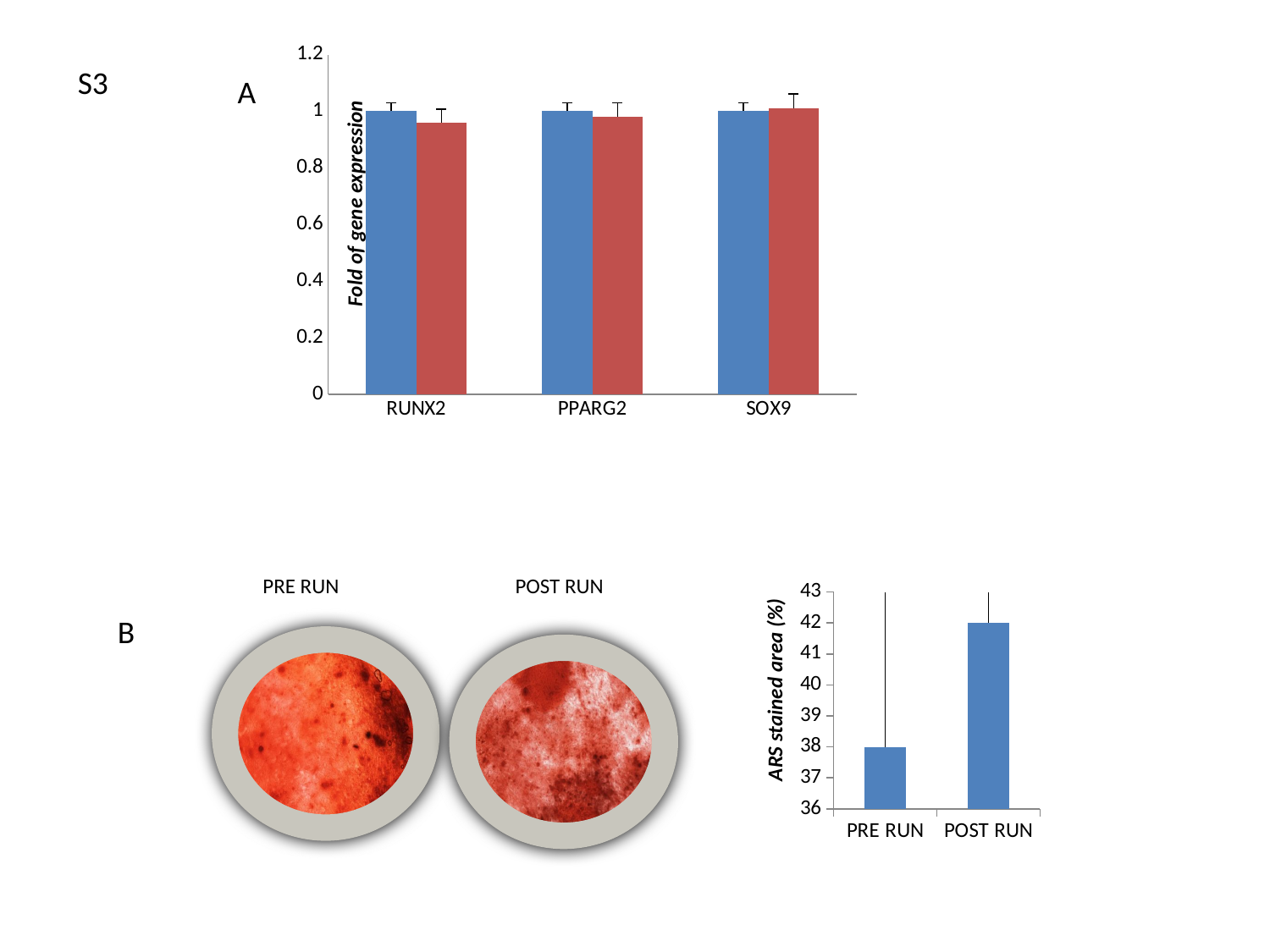

### Chart
| Category | PRE RUN | POST RUN |
|---|---|---|
| RUNX2 | 1.0 | 0.9600000000000005 |
| PPARG2 | 1.0 | 0.98 |
| SOX9 | 1.0 | 1.01 |A
Fold of gene expression
PRE RUN
POST RUN
### Chart
| Category | ARS stained area% |
|---|---|
| PRE RUN | 38.0 |
| POST RUN | 42.0 |ARS stained area (%)
B
S3

## Slide 4
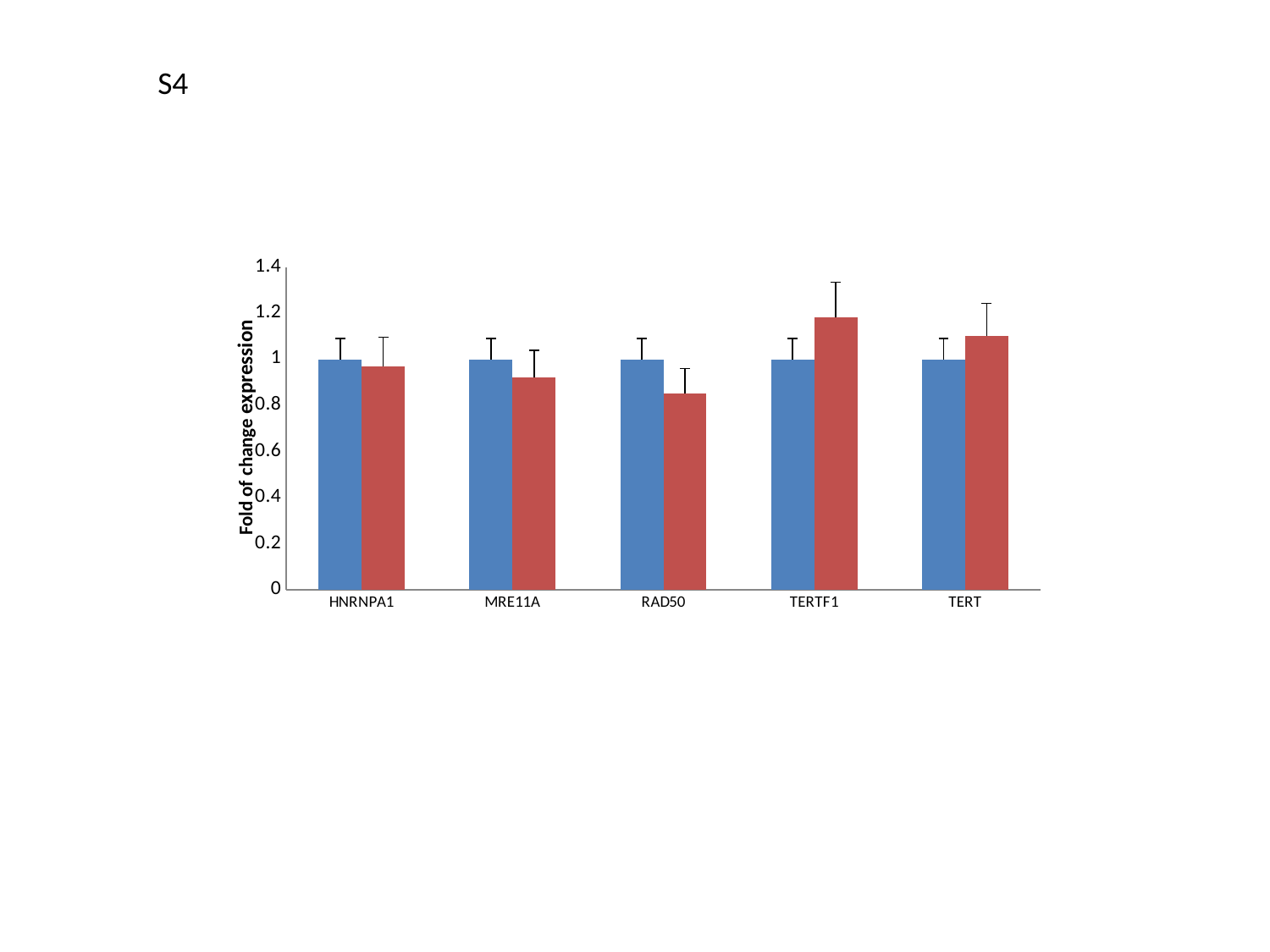

S4
### Chart
| Category | CONTROLS PRE | CONTROLS POST |
|---|---|---|
| HNRNPA1 | 1.0 | 0.9700000000000003 |
| MRE11A | 1.0 | 0.92 |
| RAD50 | 1.0 | 0.8500000000000004 |
| TERTF1 | 1.0 | 1.1800000000000008 |
| TERT | 1.0 | 1.1 |Fold of change expression

## Slide 5
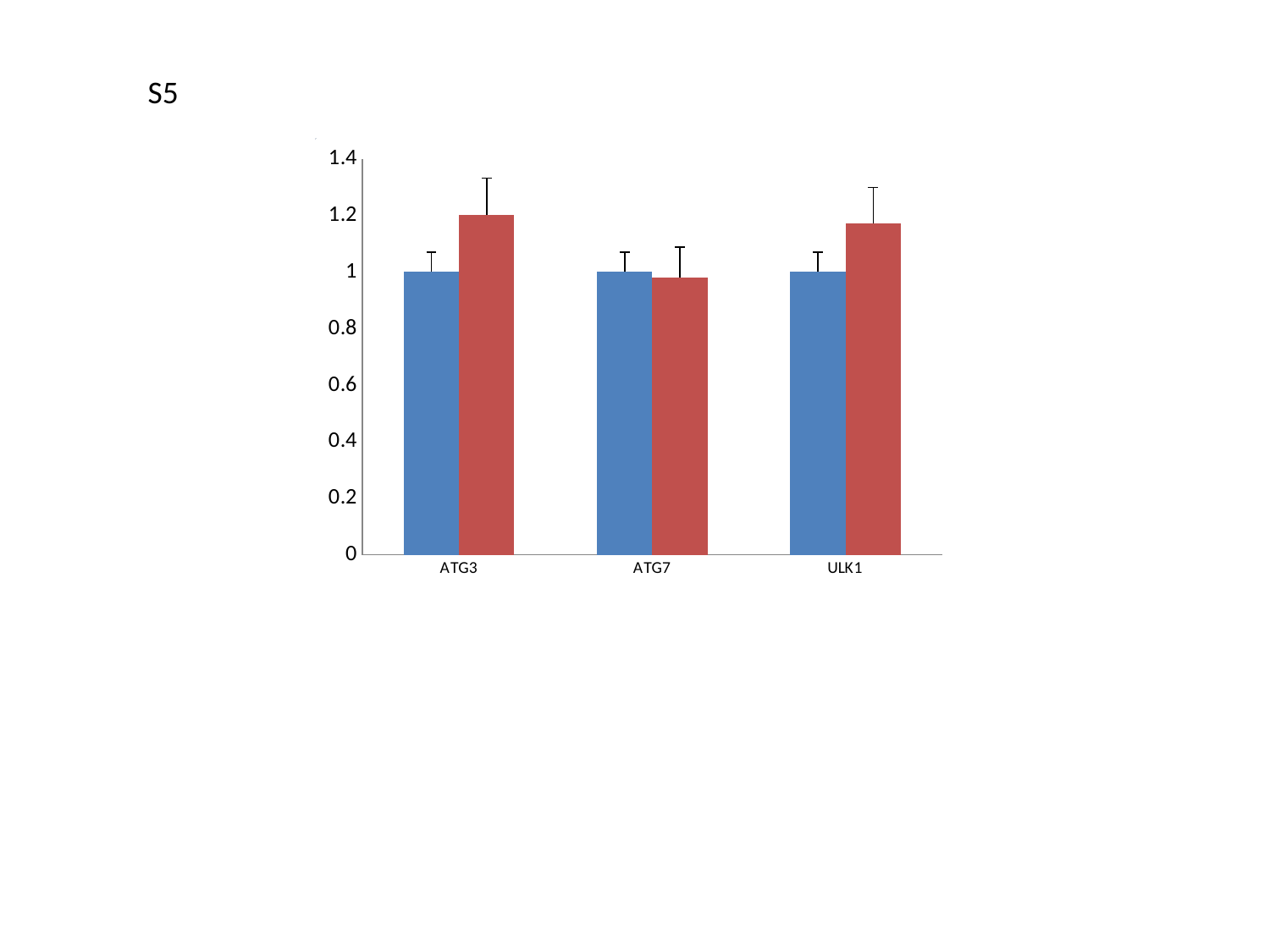

S5
### Chart
| Category | CONTROLS PRE | CONTROLS POST |
|---|---|---|
| ATG3 | 1.0 | 1.2 |
| ATG7 | 1.0 | 0.98 |
| ULK1 | 1.0 | 1.1700000000000008 |
