# Supplementary material for: Increased Gene Expression of RUNX2 and SOX9 in Mesenchymal Circulating Progenitors Is Associated with Autophagy during Physical Activity
Source: Oxid Med Cell Longev. 2019 Oct 15;2019:8426259. doi: 10.1155/2019/8426259 (PMC6815530; doi:10.1155/2019/8426259)
Supplement: Supplementary Materials — Supplemental Table 1: osteogenic Array analyses. Supplemental Table 2: telomerase related genes analyses. Figure 1S: RT real-time PCR data: expression of osteogenic genes in control samples obtained at time 0 and after 2 hrs. Figure 2S: RT real-time PCR data: expression of adipogenic genes in control samples obtained at time 0 and after 2 hrs. Figure 3S: expression of osteogenic (RUNX2), adipogenic (PPARG2), or chondrogenic (SOX9) transcription factors (A) and Alizarin red staining (B) in the MSC line treated with control sera. Figure 4S: RT real-time PCR data: expression of telomerase-related genes in control samples obtained at time 0 and after 2 hrs. Figure 5S: RT real-time PCR data: expression of autophagy-related genes in control samples obtained at time 0 and after 2 hrs. [file 8426259.f1.zip › Supplemental Table 1.docx]

**Supplemental Table 1:** Osteogenic array; Fold change of gene expression of POST RUN vs PRE RUN

| **Gene Symbol** | **Fold Change** | **Gene Symbol** | **Fold Change** |
| --- | --- | --- | --- |
| ALPL | 8.0±2.7* | MGP | 3.8±1.5* |
| ARSE | 1.4±0.5 | MINPP1 | 1.0±0.8 |
| BGLAP | 0.8±0.2 | MMP2 | 1.3±1 |
| BMP1 | 0.6±0.2* | MMP8 | 1.9±1.4* |
| BMP2 | 1.3±0.8 | MSX1 | 4.9±2.9* |
| BMP3 | 2.2±1* | MSX2 | 0.3±0.1* |
| BMP4 | 0.4±0.1* | PDGFA | 2.0±1.2* |
| BMP6 | 1.9±1.2* | PHEX | 1.8±1.2 |
| BMPR1A | 0.4±0.2 | RUNX2 | 3.8±1.9* |
| CALCR | 2.6±1.4* | SMAD1 | 1.6±1* |
| COL10A1 | 1.3±0.8 | SMAD2 | 1.4±1.1 |
| COL15A1 | 2.3±1.5* | SMAD3 | 0.9±0.3 |
| COL16A1 | 1.3±0.7 | SMAD4 | 0.8±0.2 |
| COL18A1 | 1.3±0.8 | SMAD5 | 1.1±0.6 |
| COL19A1 | 0.4±0.1 | SMAD6 | 0.9±0.3 |
| COL1A2 | 2.4±1.4* | SMAD7 | 0.4±0.1* |
| COL4A3 | 0.8±0.2 | SMAD9 | 0.7±0.1 |
| COL4A4 | 1.8±1.0* | SOST | 1.2±0.9 |
| COL5A1 | 1.4±0.9 | SOX9 | 1.8±1.2* |
| COL7A1 | 3.7±1.8* | SPARC | 2.3±1.5* |
| COL9A2 | 1.1±0.7 | SPP1 | 3.3±1.4* |
| COMP | 1.9±1.2* | TFIP11 | 0.6±0.2* |
| CSF2 | 0.8±0.5 | TGFB1 | 1.6±1.2 |
| EGF | 2.6±1.1* | TGFB2 | 2.3±1.3* |
| EGFR | 0.3±0.1* | TGFB3 | 0.9±0.3 |
| ENAM | 1.83±1.2 | TGFBR1 | 0.8±0.1 |
| FGF2 | 2.6±1.6* | TGFBR2 | 1.0±0.2 |
| FGFR1 | 1.3±0.8 | TUFT1 | 0.6±0.1* |
| FGFR2 | 0.3±0.1* | TWIST1 | 2.5±1.4* |
| FGFR3 | 1.8±1.1 | TWIST2 | 1.0±0.3 |
| FLT1 | 2.9±1.6* | VDR | 1.3±0.2 |
| IGF1 | 0.4±0.1* | VEGFA | 1.1±0.5 |
| IGF1R | 1.1±0.6 | VEGFB | 1.1±0.2 |
| IGF2 | 4.4±2.5* | VEGFC | 2.7±1.3* |

*p> 0.05
