# Supplementary material for: Increased Gene Expression of RUNX2 and SOX9 in Mesenchymal Circulating Progenitors Is Associated with Autophagy during Physical Activity
Source: Oxid Med Cell Longev. 2019 Oct 15;2019:8426259. doi: 10.1155/2019/8426259 (PMC6815530; doi:10.1155/2019/8426259)
Supplement: Supplementary Materials — Supplemental Table 1: osteogenic Array analyses. Supplemental Table 2: telomerase related genes analyses. Figure 1S: RT real-time PCR data: expression of osteogenic genes in control samples obtained at time 0 and after 2 hrs. Figure 2S: RT real-time PCR data: expression of adipogenic genes in control samples obtained at time 0 and after 2 hrs. Figure 3S: expression of osteogenic (RUNX2), adipogenic (PPARG2), or chondrogenic (SOX9) transcription factors (A) and Alizarin red staining (B) in the MSC line treated with control sera. Figure 4S: RT real-time PCR data: expression of telomerase-related genes in control samples obtained at time 0 and after 2 hrs. Figure 5S: RT real-time PCR data: expression of autophagy-related genes in control samples obtained at time 0 and after 2 hrs. [file 8426259.f1.zip › Supplemental Table 2.docx]

**Supplemental Table 2**: Telomerase-associated genes array; Fold change of genes expression in pooled POST RUN vs PRE RUN M-CPCs

| GENE SYMBOL | FOLD CHANGE |
| --- | --- |
| HNRNPA1 | 0.5±0.1* |
| HNRNPA2B1 | 1.2±0.6 |
| HNRNPAB | 1.0±0.3 |
| HNRNPC | 1.1±0.2 |
| HNRNPD | 1.4±0.5 |
| HNRNPF | 0.9±0.2 |
| MRE11A | 0.9±0.3 |
| NBN | 0.6±0.1* |
| POT1 | 0.6±0.2 |
| RAD50 | 0.5±0.1* |
| TERF1 | 6.4±2.3* |
| TERF2 | 0.8±0.2 |
| TERF2IP | 1.1±0.7 |
| TERT | 3.5±1.2* |
| TINF2 | 0.8±0.2 |
| TNKS | 1.0±0.1 |
| TNKS2 | 0.9±0.1 |
| XRCC5 | 0.9±0.05 |
| XRCC6 | 0.9±0.1 |

***p<0.05**
